# Targeting Iron Responsive Elements (IREs) of APP mRNA into Novel Therapeutics to Control the Translation of Amyloid-β Precursor Protein in Alzheimer’s Disease

**DOI:** 10.3390/ph17121669

**Published:** 2024-12-11

**Authors:** Mateen A. Khan

**Affiliations:** Department of Life Science, College of Science and General Studies, Alfaisal University, Riyadh 11533, Saudi Arabia; matkhan@alfaisal.edu

**Keywords:** Alzheimer’s disease, amyloid precursor protein, iron regulatory protein, iron responsive elements, iron

## Abstract

The hallmark of Alzheimer’s disease (AD) is the buildup of amyloid-β (Aβ), which is produced when the amyloid precursor protein (APP) misfolds and deposits as neurotoxic plaques in the brain. A functional iron responsive element (IRE) RNA stem loop is encoded by the APP 5′-UTR and may be a target for regulating the production of Alzheimer’s amyloid precursor protein. Since modifying Aβ protein expression can give anti-amyloid efficacy and protective brain iron balance, targeted regulation of amyloid protein synthesis through modulation of 5′-UTR sequence function is a novel method for the prospective therapy of Alzheimer’s disease. Numerous mRNA interference strategies target the 2D RNA structure, even though messenger RNAs like tRNAs and rRNAs can fold into complex, three-dimensional structures, adding even another level of complexity. The IRE family is among the few known 3D mRNA regulatory elements. This review seeks to describe the structural and functional aspects of IREs in transcripts, including that of the amyloid precursor protein, that are relevant to neurodegenerative diseases, including AD. The mRNAs encoding the proteins involved in iron metabolism are controlled by this family of similar base sequences. Like ferritin IRE RNA in their 5′-UTR, iron controls the production of APP in their 5′-UTR. Iron misregulation by iron regulatory proteins (IRPs) can also be investigated and contrasted using measurements of the expression levels of tau production, Aβ, and APP. The development of AD is aided by iron binding to Aβ, which promotes Aβ aggregation. The development of small chemical therapeutics to control IRE-modulated expression of APP is increasingly thought to target messenger RNAs. Thus, IRE-modulated APP expression in AD has important therapeutic implications by targeting mRNA structures.

## 1. Introduction

The most prevalent neurodegenerative disease, Alzheimer’s disease (AD), causes a progressive deterioration in cognitive function and is characterized by pathological features such as the production of neurofibrillary tangles (NFT), amyloid-β (Aβ) protein, and senile plaque. Aβ-protein buildup in brain areas that are crucial for memory and cognition is aberrant in AD. The amyloid precursor protein (APP) is the source of Aβ, a typical by-product of cellular metabolism. Conversely, an abundance of physiologically significant pathways leading to the death of neurons are adversely affected by excess production of Aβ, as well as by its aggregation and deposition. The Aβ is produced when β- and γ-secretases break APP at the plasma membrane in succession. Either the freshly created Aβ-peptide is discharged into the extracellular space, or it stays connected to the lipid raft structures and plasma membrane [1,2,3]. Aggregation of Aβ-peptide is significantly favored by its binding to ganglioside in the lipid rafts [4]. Aβ fibrils have been described as the neurotoxic substance responsible for progression of AD [5]. Over recent decades, it has been indicated that the Aβ peptide’s oligomeric or prefibrillar forms do the greatest harm to neural cells. Dementia can arise from the binding of soluble Aβ to various substances in the extracellular space, such as metals, cell surface receptors, and central nervous system cellular membranes [6,7,8]. Extracellular Aβ peptide aggregation, intracellular hyperphosphorylated tau protein buildup in NFT formations, and an increased iron concentration in the brain are the predominant pathological features characterizing AD [9,10,11]. Numerous genes implicated in cellular iron metabolism, amyloid overexpression, and neuronal cell death have been linked to recent findings [12,13]. These findings show a clear connection between abnormal iron homeostasis and amyloid accumulation in the brains of AD patients.

Iron dysregulation in the brain is associated with several neurodegenerative diseases, including AD and Parkinson’s disease (PD) [14]. It is known that a misregulation of iron in the brain is one of the main causes of neuronal death in AD [15,16]. Abnormalities of iron metabolism in neurons and/or astrocytes are directly linked with amyloid overexpression [17,18]. It has been demonstrated that neurodegenerative illness can worsen when the iron steady state in neurons is disturbed [19,20,21,22]. Iron overload in the brain tissues causes abnormalities in the IRP/IRE signaling system, which in turn causes an excess of amyloid, its aggregation, the loss of neurons, and the advancement of AD [23]. Iron is known to play a significant role in the neurotoxicity associated with AD and PD, as evidenced by the presence of 5′-untranslated region (5′-UTR) IRE sequences in the transcripts of the APP and α-synuclein (α-syn), the activity of APP as a neuronal iron export ferroxidase, and the ability of iron to increase the neurotoxicity of Aβ [24]. Iron regulating protein is the primary modulator of intracellular iron homeostasis. Through specific binding to the conserved IREs found in the UTRs of mRNAs, it post-transcriptionally regulates many genes involved in iron homeostasis [25,26]. The IRE stem loop in the APP transcript indicates that this protein is important in iron homeostasis, as other iron-regulated proteins have shown. The inability to control iron binding has an impact on how IRPs interact with IREs [27], which can have an impact on Alzheimer’s amyloid overexpression. The mis-regulation of these essential proteins during AD may be attributed in part to the shape of the IRE RNA stem loop. Given that iron regulates intracellular protein production, the impact of intracellular iron levels on the APP 5′-UTR makes sense [28,29].

**Figure 1:**
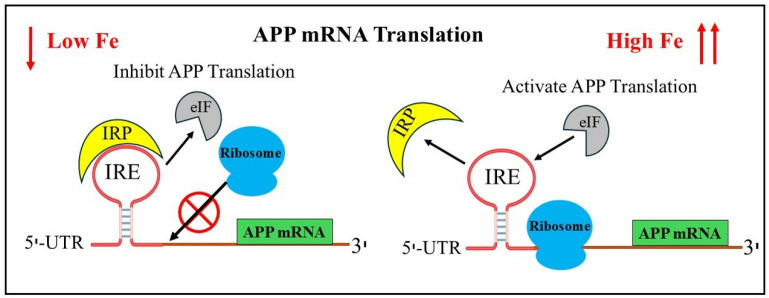
Proposed model for iron-induced translation modulation by the IRP/IRE signaling pathway. Low cellular iron promotes the binding of IRP, release of eIF4F and blocking ribosome binding to IRE in the UTRs of APP thus preventing APP translation. In cellular iron overload, iron displaces IRPs from the IRE mRNA. IRP1 dissociates, allowing ribosome and initiation factor eIF4F binding and translation of APP proceeds. Black arrows showing either eIF/IRP interacting or dissociating from IRE stem-loop structure of RNA. Red cross means blocking of ribosome binding.

Iron responsive elements (IREs) can be present in either the 5′- or 3′-UTR of the target APP mRNA [30]. IREs fold into stem loop structures, which are bent A-RNA helices with terminal loops [30,31]. The protein IRPs are tightly bound to the IRE RNA structures. Misregulated binding of IRPs to IREs in excess brain iron is expected to have an impact on the expression of IRE-controlled genes [27]. During AD, deregulation of these essential proteins may be triggered by the general IRE RNA stem loop. The structure of the IRP is altered by the binding of the APP IRE RNA. The paired and unpaired bases of each IRE RNA correlate to the IRP binding stabilities, which differ by a factor of ten within the IRE family. The in vivo graded iron responses are impacted by this variance [32]. Changes in the intracellular iron concentrations either stabilize or destabilize the IRP/IRE complex. IRPs interact with the IRE stem loop structure, which in turn affects APP expression [33]. Figure 1 shows that the specific translation regulation pathways of the IRE/IRP interactions in the 5′-UTR modulate the expression of the mRNA transcript encoding APP, which is responsive to cellular iron levels. IRP has the ability to function as a translational inhibitor or enhancer [23]. IRP1 binds to the APP IRE with greater affinity as iron levels fall, repressing the translation of the APP protein. The fraction of mRNA in IRE RNA/IRP1 complexes, which prevent ribosome binding, decreases and the fraction of mRNAs in polyribosomes increases when iron concentrations in cells rise. This is because mRNAs with three-dimensional noncoding structures in the 5′-UTRs are derepressed [34]. IRP1 is released from IRE RNA, which is found inside the 5′-UTR APP mRNA, by free ferrous iron, which results in increased protein synthesis.

The necessary conformational shift that IRPs must undergo to bind IRE RNA is demonstrated by the interactions between IRE-binding protein and cytosolic aconitase. This necessitates significant conformational and domain movements of IRP in the RNA-binding pocket mechanism. These alterations in IRP1 structural conformation following binding to IRE were detected by X-ray crystallography and circular dichroism (CD) spectroscopy [35,36]. The structural changes that arise when eukaryotic translation initiation factors (eIFs) bind to the mRNA cap moiety and IRE RNA were explained using similar methods [37,38]. The relationship between iron homeostasis and the expression of amyloid via APP IRE 5′-UTR mRNA, as well as the possible therapeutic and clinical uses of these molecules in AD, are summarized here. In addition, I observed that several IREs can bind to IRPs by examining the set of RNA sequences that make up the IRE family. These reports demonstrated the significance of the pseudotriloop conformation of APP and ferritin IRE for binding to IRPs by using the biophysical and computational techniques [38,39,40] and the yeast three-hybrid system [32], which has been widely used to assay protein–RNA interactions in vitro [41,42] and in vivo [43]. This feature article aims to explain the biology of iron and the IRE in the 5′-UTR APP mRNAs in terms of its structure and function, while also referring to the physiological role of iron in promoting the aggregation of the Aβ-peptide and outlining iron’s role towards promoting in-depth toxic effects of APP processing during the etiology of AD. Additionally, this report highlights the advancements in small molecular compounds that can target APP 5′-UTR mRNA to reduce APP expression with therapeutic implications in AD.

## 2. Iron Homeostasis in Brain

Iron is involved in many physiological processes in the brain, including respiration, oxygen transport, DNA synthesis, energy metabolism, neuronal myelination, and neurotransmitter production; maintaining the proper physiological level of iron in the brain is essential [44]. Iron enters the brain either bound to transferrin (Tf), which allows iron to pass through the blood–brain barrier or the blood–cerebrospinal fluid barrier, or possibly unbound, particularly in circumstances where transferrin gets saturated with iron and causes iron overload [45]. Iron is the most prevalent metal ion in the central nervous system’s glial and neuronal cells. Since transferrin receptor 1 (TfR1) has only been documented in vitro thus far, it is believed that non-Tf bound iron [46] is the primary mediator of iron uptake into astrocytes [47]. The relationship between astrocytes and endothelial cells controls the integration and transport of iron inside the brain. There is no TfR1 on astrocytes. TfR1 binds ferric iron (Fe^3+^)-loaded Tf in the luminal membrane of endothelial cells. The endosomal compartment internalizes this complex and converts Fe^3+^ to ferrous iron (Fe^2+^) [48]. TfR1 is a protein that has a high affinity for Fe^2+^ Tf [49]. It combines with the TfR2 to generate a compound that the endosomal compartment then absorbs. Then, Fe^3+^ is reduced to Fe^2+^ [34], enabling divalent metal transporter-1 (DMT1) to transport the complex across the endosomal membrane and into the cytosol [50,51]. Apo-Tf binds to the Tf receptor very firmly in the acidic endosome. The breakdown of free Tf is stopped by this contact before exocytosis, which happens when the endosome fuses with the lysosome. When the pH becomes neutral again after exocytosis, the apo-Tf separates from the TfR1 and recycles the Tf molecule for additional use in the iron cycle. Since neurons express both DMT1 and TfR1, the process by which they take up iron is receptor-mediated endocytosis [52,53].

Excess iron is stored in the cytoplasmic labile iron pool and mitochondria for the synthesis of iron–sulfur (Fe-S) clusters and heme prosthetic groups to the cytoplasmic iron storage protein ferritin, and to the bloodstream by Fpn1. This process occurs after excess iron enters the cytoplasm of these cells and satisfies metabolic requirements [48]. In mammalian brain cells, Fpn mediates the only known export pathway [54]. Fpn facilitates the cell’s export of Fe^2+^; however, for Tf to bind the iron, ferroxidase must change the exported iron into Fe^3+^. It has been observed that Fpn and its associated ferroxidases, such as APP, are expressed by brain astrocytes and neurons. The ferritin L-subunit, which oversees maintaining long-term iron storage, and the ferritin H-subunit, which has a ferroxidase activity to promote the quick absorption and utilization of iron, are the two subunits of the iron storage protein ferritin. Although ferritin-H is more widely distributed and the ratio of H-to-L-ferritin varies on whether regional cells consume iron, both subunits are expressed throughout the brain [55]. Ferritin is the primary protein, found in glial cells but absent in neurons, that buffers and stores iron [56]. Iron and other metals have been shown to accumulate in the choroid plexus [57], and iron is thought to be a metal of intermediate toxicity in terms of its capacity to damage the blood–brain barrier and the blood–cerebrospinal fluid barrier [57,58]. Any disruption of homeostasis, such as genetic variations impacting metal excretion or increased exposure to iron in the environment [59], may permit the build-up of iron, thereby making neurodegeneration more likely. Neuromelanin (NM) is stored by neurons in human brain regions such the substantia nigra (SN) and locus coeruleus (LC). This NM is subsequently used to sequester excess labile iron [21]. SN is frequently affected by early neurodegenerative illnesses, and it is noteworthy that SN is impacted by AD, PD, and LC [60,61].

Protein aggregates, a feature that sets neurodegenerative diseases apart, appear to draw iron and other metals, which function as glue and cause misfolding of proteins. Iron overload in the brain accumulate inside and around Aβ fibrils and Aβ plaques in AD [27]. Iron levels in the amyloid plaques were shown to be higher in the Tg2576 mouse model of AD, which is similar to what is observed in the brains of AD patients [62]. It is interesting to note that APP, which causes AD, has been connected to peculiar IRPs [63,64]. Therefore, APP translation may be triggered by elevated cytosolic iron. The exact connection between iron and AD onset is yet unknown, although iron levels in the body must be carefully regulated. These findings suggest that iron encourages accumulation of Aβ plaques in the brain cells. The accumulation of iron causes degeneration of neurons and glial cells, as well as neuroinflammation and immune cell infiltration at the locations of small lesions [65,66]. Higher iron levels in AD brains have been linked to higher ferritin levels in the surrounding neuroglia cells [67]. Iron is known to cause amyloidosis and Aβ peptide aggregation, and in cultured cells, Aβ peptide complexed with iron is toxic to the nerve fibers in the central nervous system [68].

In addition to amyloid plaque, neurodegenerative dementia and Lewy body disease can also be modelled by the path that leads to accelerated disruptions of iron homeostasis and ferroptosis as causes of neuronal death [69]. Ferroptosis is an iron-regulated necrosis that is distinct from apoptosis in that it is characterized by condensed or shrunken mitochondria brought on by an excess of intracellular redox active iron and the lack of it [70]. This ferroptosis is linked to elevated redox active lipids in neurons and pancreatic cells in conditions including AD, PD, and diabetes [69]. By disrupting the IRE-dependent translation of ferritin and APP, the environmental metallotoxins lead (Pb), iron (Fe), and manganese (Mn) kill neurons. Because of the excess unsorted intracellular iron and the production of harmful ROS, the loss of these iron homeostatic neuroprotectants led to an embargo on iron export from neurons, which in turn enhanced cellular redox and ROS-induced neuronal death. These events are linked to ferroptosis and are known to be enhanced by iron catalyzing the production of hydroxyl free radicals [71].

Ferroptosis and morphological alterations to mitochondria have been connected to α-syn-associated iron accumulation in PD as an early preapoptotic event in the neurodegeneration of dopaminergic neurons [72]. Through shared mechanisms that toxically accelerate IRP/IRE disruption of ferritin translation and intracellular iron homeostasis to cause ferroptosis, excess brain iron causes neurons to be less lethal than they should be. Glutathione, on the other hand, functions as an opposing antioxidant [23]. The 5′-UTR of the α-syn transcript was anticipated to encode an iron responsive region, like that of APP mRNA [32]. The α-syn, prion protein, and APP’s functions in iron transport have been connected to the IRE/IRP-dependent ferritin regulatory pathways of translation. When cells are exposed to excessive iron in a neurotoxic manner, several pathways work together to produce ferroptosis. As with amyloidogenesis in AD and Lewy body forms through α-synucleinopathy in PD, ferroptosis undoubtedly compromises neuronal survival and is linked to neurodegenerative diseases of cognitive decline [69].

## 3. Iron Dysregulation in Alzheimer’s Disease

Dysregulation of iron homeostasis in the brain has been implicated in the pathogenesis and progression of AD [14,73]. Elevated iron levels may facilitate the onset and advancement of illness by co-localizing with Aβ plaques. Excess iron concentration build-up has been shown in the vicinity of amyloid plaques and NFTs [74], which accelerate the development of Aβ plaques [75,76]. Iron regulates the processing of APP via interactions between APP IRE and IRPs, which suggests the involvement of APP holoprotein in iron metabolism [77,78]. Increased APP altered brain iron homeostasis based on its capacity to bind Fpn and export iron. It has been reported that familial Alzheimer’s disease (FAD) can be the result of a genetically inherited overexpression of the APP gene that causes APP to be overexpressed and increases the risk of AD by downregulating the effects of iron on brain function and ferritin, a protein involved in iron homeostasis [79]. APP IRE mRNA can indirectly regulate neuronal iron levels and promote iron export by maintaining the stability of Fpn [28]. Tau proteins have the ability to stabilize the Fpn1-APP complex and are also capable of delivering APP to the cell membrane [80].

**Figure 2:**
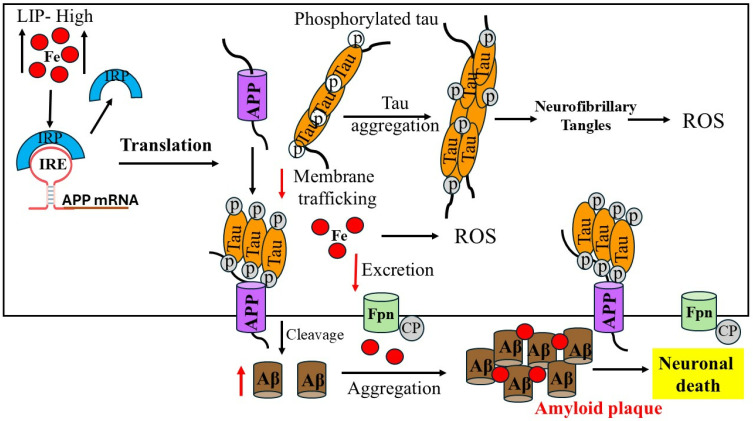
Alzheimer’s disease with iron dysregulation in the brain. How the production of APP and the promotion of amyloid plaques in AD are caused by an increase in the labile iron pool (LIP) in neurons.

Additionally, iron itself stimulates the development of oligomeric tau, and an excess of iron causes the tau protein to be hyperphosphorylated [81]. With a focus on iron’s ability to bind to Aβ-peptide and increase Aβ toxicity, Figure 2 illustrates the process of brain iron dysregulation and its relationship to AD. It is believed that a metabolic event that happens in AD individuals is produced by an increase in the labile iron pool of neurons in young, healthy individuals. Iron regulatory proteins are essential for controlling the uptake, storage, and excretion of proteins by neuronal cells in response to an increase in the labile iron pool in healthy individuals. As a result, increased labile iron pool, iron export and storage are made possible by higher ferritin and APP production, respectively. Conversely, reduced expression of TfR1 and DMT1 inhibits iron import. By lowering the amount of soluble tau, hyperphosphorylation and tau aggregation prevent APP from being transported to the cell membrane, which accelerates the production of labile iron in neurons [82,83]. Another feature of AD is increased APP cleavage into Aβ, which assembles into amyloid plaques due to its strong affinity for iron and causes synapse loss and neuronal death. Moreover, neurodegeneration results from the iron buildup in AD neurons depleting the iron reserves of other brain cells. Iron influx drives the translational expression of the neuronal APP, which is strongly associated with the interaction of APP IREs in the 5′-UTR mRNA and IRP1, allowing cellular iron levels to control APP translation directly [72].

The ability of the transmembrane protein APP to disintegrate Aβ has been the subject of much research, despite its complex roles in iron transport, synapse formation, and brain development [84]. Under normal settings, the majority of neuronal APP is cleaved by proteolytic means using α- and β-secretases, which are part of the non-amyloidogenic route. The amyloidogenic process cleaves APP first with β-secretase and then with γ-secretase to create Aβ peptides. Because iron levels regulate APP expression at the translational level, increased iron concentrations in neurons increase the amount of APP expression that can be processed amyloidogenically, thereby speeding up the synthesis of Aβ. Deletion of APP results in internal iron retention in mice models and cultured neurons. Further, APP promotes iron efflux from neurons through its stabilizing interaction with surface Fpn [85,86]. When tau is absent, APP cannot migrate to the membrane, connect with Fpn, and stop iron from leaving neurons. Iron can have an impact on how APP is processed; at high concentrations, iron prevents APP from growing while maintaining immature APP. The primary component of amyloid plaques is the 40–42 amino acid Aβ peptide, which is created by proteolysis of a single substrate called APP [87]. Among the ultrastructural prerequisites required for the polymerization of Aβ peptide is iron [88]. Elevated iron levels have been demonstrated to accelerate Aβ activity, which stimulates the Aβ-induced cell death of neuroblastoma.

A direct correlation between elevated iron levels and the decline in neuronal function has been observed in individuals with AD. The altered expression of iron regulatory proteins suggests an iron misregulation in AD brains. Protein that is significantly enhanced in AD patients is iron storage ferritin, which is directly correlated with enhanced iron level. It has been reported that amyloid plaques contain increased quantities of iron and ferritin-rich cells in the postmortem brain samples of AD patients [56]. These reports suggested that the etiology of AD is associated with the misregulation of iron trafficking and its accumulation in the brain cells [89]. Iron is a pathogenic regulator of amyloid toxicity and APP translation from a mechanical point of view. Thus, changes in intracellular iron concentrations can interfere with IRPs binding to IRE RNA stem loops, affect the transferrin–ferritin balance and having an effect on the iron export protein APP [33]. These findings support the roles of transferrin genes and hemochromatosis as hereditary variables that may raise the risk of late-onset sporadic AD. When combined, iron can influence the course of AD by controlling the translational expression of APP, Aβ proteins, and tau hyperphosphorylation. Aβ-peptides first oligomerize into different soluble species then convert their confirmation into protofibrils and cross-β-sheet fibrils, forming amyloid plaques. Aβ aggregates interact with tau proteins to exert toxic effects. More research is needed to understand the connection between APP overexpression, plaque formation and the role of iron in the pathophysiology of AD.

## 4. Iron-Induced Amyloid-β Aggregation

**Figure 3:**
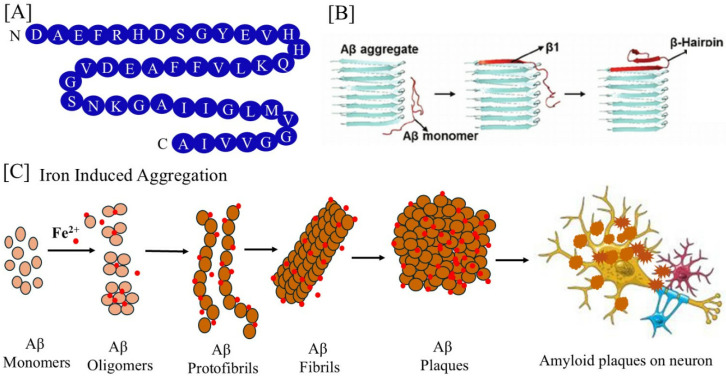
Amyloid β configuration and accumulation. (**A**) The Aβ_42_ primary amino acid sequence. (**B**) Amyloid-β peptide structures interact with Aβ aggregates during the elongation process (modified from Ref. [97]). (**C**) The aggregation of monomers of Aβ into higher-order oligomers, fibrils, protofibrils, and amyloid plaques is triggered by iron.

AD is characterized by excess build-up of the amyloid plaques on the neuron. In cerebral amyloid angiopathy, Aβ can also form deposits along the inside of cerebral blood vessels. APP, which is significantly bigger, is broken down into Aβ peptides. The most common isoforms include Aβ_1–40/42_ [90], found in the brains of AD patients. Aβ is mostly found in brain interstitial fluid, plasma, and cerebrospinal fluid as soluble Aβ_40_. In a healthy brain, the majority of Aβ consists of Aβ_40_, whereas in a sick state, extra Aβ_42_ is created and primarily accumulates as amyloid plaques. Amyloid plaques contain both Aβ_40_ and Aβ_42_ [91], while vascular amyloid is predominantly the shorter Aβ_40_. The pathophysiology of both familial and sporadic AD has been linked to increases in overall Aβ levels or the relative amounts of Aβ_40_, which is more concentrated in cerebrovascular plaques, and Aβ_42_, which is concentrated in neuritic plaques [92]. Compared to Aβ_40_, Aβ_42_ exhibits quicker aggregation kinetics and severe neurotoxicity [93]. The peptide Aβ_42_ is the most amyloidogenic because of its increased hydrophobicity. The plaques are composed of a tangle of Aβ oligomers [94] and regularly ordered aggregates called amyloid fibrils. Aβ molecules can clump together to create flexible, soluble oligomers that can take on several shapes (Figure 3). Aβ monomers combine to form oligomers, protofibrils, and amyloid fibrils, among other kinds of assemblies.

Figure 3A displays the Aβ_42_ amino acid sequence. From low-molecular-weight oligomers such as dimers, trimers, tetramers, and pentamers to higher-order assemblies such as hexamers, nonamers, and dodecamers to protofibrils and fibrils, Aβ monomers can be formed (Figure 3C). Moreover, partially folded units create a paranucleus by hydrogen bonding and hydrophobic contact, which then self-assembles to produce higher-order structures known as protofibrils. During the elongation phase, protofibrils continue to self-assemble to create lengthy fibrillar aggregates [95]. According to the molecular dynamics simulation studies, the N-terminal binds to the core Aβ fibril during elongation by intermolecular hydrogen bonding (β1) (Figure 3B) [96,97]. Whereas amyloid oligomers are soluble and have the potential to spread throughout the brain, amyloid fibrils are bigger, insoluble, and can further assemble into amyloid plaques that are indicative of AD. The Aβ oligomers have a heterogeneous size distribution. The soluble peptide takes on a collapsed coil form instead of an α-helical or β-sheet shape [98]. According to microsecond all-atom MD simulations, a certain conformation that results in ring-shaped pentamers and hexamers is stable [99]. Given that they both seem to have extended or β-sheet structures and exhibit comparable levels of main chain hydrogen bonding that is exchange-resistant, there may be some structural similarities between them. However, amyloid fibrils and oligomers seem to have non-overlapping, mutually exclusive conformations identified as generic antibody epitopes shared by amyloids with various sequences [100]. When fibrils are first developing, oligomers are a kinetic intermediate waxing. Many forms of soluble amyloid oligomers share a common structure and toxicity mechanism [101].

Additionally, it is unclear if the oligomer structures are merely in equilibrium with the monomers, which immediately form fibrils without the need for an intermediary oligomeric structure, or if they represent the building blocks of amyloid protein that eventually assemble into fibrils. Early in the process, oligomers take the shape of spherical aggregates. Later, they elongate by coalescing spherical subunits into beads, which forms the precursor of protofibrils on the route to mature fibers. One important organizing factor for amyloid oligomers is the parallelism between Aβ monomers, which may also act as a shared structural motif for amyloid fibrils. Despite significant advancements, the aggregation routes and Aβ aggregate types continue to be difficult research problems. Potential pathogenic interactions and structural implications have been uncovered by the interactions between Aβ-oligomers and iron.

Transition metals like Fe^2+^, Cu^2+^, and Zn^2+^ can be bound by oligomers that are typically imbedded in the membrane [102]. As a result, Aβ peptide aggregation is accelerated in metal-bound senile plaques and metal ion homeostasis in the brain may be regulated by Aβ oligomer production [103]. Increased aggregation rates, oligomer state stabilization, and ROS formation have all been seen in the presence of excess Fe^2+^ coordination with Aβ [104]. Iron binds to Aβ, especially in plaque cores, and delays Aβ typical orderly progression to fibrils and other highly ordered aggregates. In brain cells, this iron interference increases Aβ toxicity by the oligomer’s formation [8,68]. Misfolded Aβ can cause tau to misfold, the other protein linked to AD, likewise generating these prion-like misfolded oligomers [105].

Several studies in animal models, biochemistry, genetics, cell biology, and experimental biology bolster the idea that Aβ is a key player in the pathophysiology of AD. Moreover, Aβ that is released into the extracellular space can move between various compartments, including the brain and blood, as well as be cleared by chaperones like apoE. These chaperones can impact Aβ metabolism subsequent to cell release and can also affect Aβ aggregation, clearance, and transport [106]. Amyloid burden and Aβ levels in the central nervous system can be decreased by techniques to remove Aβ from the circulatory system. Therefore, treatments modulate to lower Aβ aggregation by lowering Aβ synthesis and APP translation. Through a better understanding of the structures and aggregation routes, novel treatment may be developed to inhibit the development of toxic aggregates.

## 5. Alzheimer’s Amyloid-β Transcript Encodes Functional IRE mRNA

**Figure 4:**
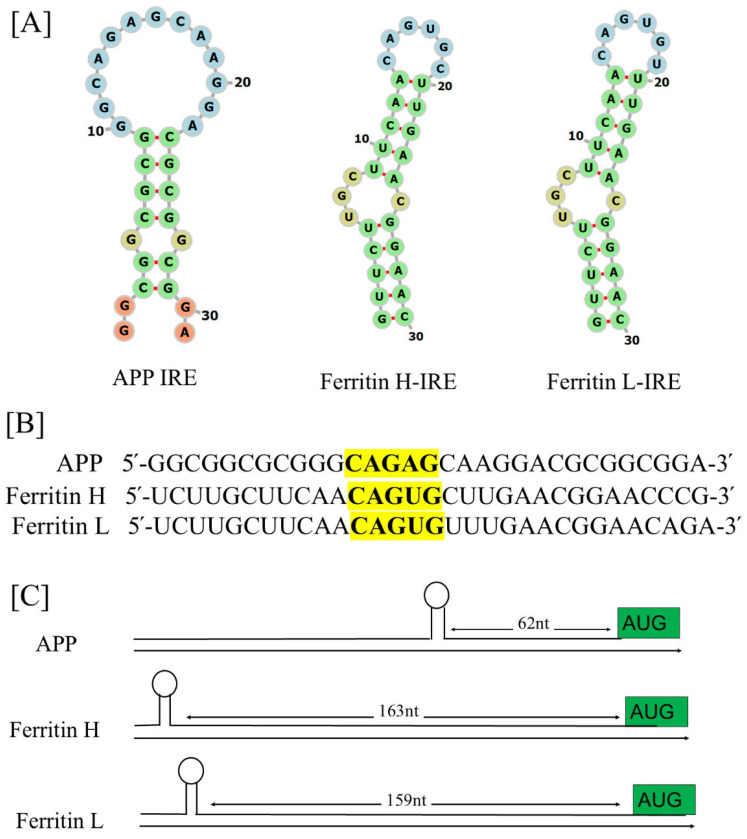
APP IRE RNA 5′-UTRs were predicted to fold into stable RNA stem loops like the 5′-UTR-specific IRE in ferritin-H and ferritin-L transcript. (**A**) APP, ferritin-H, and ferritin-L stem loop IRE structures. (**B**) After comparing sequences encoding the 5′-UTR-specific IRE stem loop with ferritin IRE (which were displayed in two clusters of greater than 70% sequence similarity), the APP IRE was discovered. Key IRE motifs are highlighted in yellow. (**C**) APP, ferritin-H, and ferritin-L transcript maps of the 5′-UTR IRE stem loops.

A functional IRE-containing mRNA has been found to be present in the 5′-UTR transcripts of Alzheimer’s APP [28,32]. A highly similar canonical loop motif is shared by the stem loops of the APP IRE RNA and the ferritin-H IRE RNA [28]. Figure 4A shows the specific IRE RNA stem loops encoded by the 5′-UTRs of transcripts linked to APP in contrast to ferritin-H and ferritin-L, the generic IRE. Each IRE-mRNA encodes uniquely configured variations of an IRE RNA that potentially bind to the IRP translational repressors in their 5′-UTRs [107]. Mammals have highly conserved individual ring (IRE) structure and sequence, which are typically composed of an apical loop motif and a stem loop element separated from a lower stem by a C-bulge [108,109,110,111]. The short (9–10 bp) double-stranded helix of all IRE RNA has an unpaired C in the middle that forms a bulge. Since all IRE RNAs construct the RNA A-helix with the identical bulge C and terminal loop sequence, the IRE sequences of various mRNAs differ very little.

Figure 4B shows the alignments encoding IRE RNA stem loops of the 5′-UTR transcripts for APP relative to the canonical ferritin H and ferritin L IRE stem loop. The ferritin 5′-UTR, which encodes a region similar to the IRE but varies from the recognized APP IRE, is one example of this homology [112]. Each mRNA encodes a different variant of an IRE RNA stem loop that can bind to the 5′-UTRs of IRP translational repressors. The binding stabilities of the APP IRE/IRP1 complex [40] and ferritin IRE/IRP1 complex [41] appear to be similar. In these 5′-UTR regions, alignments revealed that the APP and ferritin IRE RNA sequences were more than 70% identical. The projected IRP1 binding AGU/AGA tri-loops and the canonical ferritin IRE’s CAGUGN loop domain are the key features of this homology. It has been demonstrated that these tri-loops are essential for both IRP1 and IRP2 binding as well as translation suppression [32,113]. Variations in the quantities of proteins encoded in IRE mRNAs within cells are partly due to different mRNA stabilities [39,41,114]. The phylogenic conservation of a single IRE RNA (90%) is required to surpass the RNA sequence conservation of IRE RNAs expressing distinct proteins (~80%) to achieve the current array of IRP binding constants.

Figure 4C illustrates the maps of IRE RNA stem loops in the 5′-UTRs of the transcripts for APP relative to the canonical ferritin-H and ferritin-L chain IRE stem loops. It is crucial to locate the IRE inside the mRNA UTRs. The effectiveness of 5′-UTR regulation may be impacted by the IRE’s distance from the mRNA cap and AUG start site [23]. Depending on the gene type, these IREs to start and IREs to cap distances can change. On the contrary, ferritin mRNA differs significantly less from IRE RNA sequence variations in other mRNAs from the same species. APP mRNA is one example of an individual IRE RNA that is conserved across species [30]. The stem of the longer IRE RNA sequences sometimes contains extra base pairs below the bulged C. All the IRE RNAs in the 5′-UTR increase translation when iron is high, inhibit ribosome binding, and promote translation when iron is low, even though the IRE RNA sequences with a higher affinity for regulatory proteins respond to iron more quantitatively. We characterized the presence of a functional IRE stem loop structure in the 5′-UTR APP mRNA similar to ferritin IRE mRNA [40]. The sequence alignment between the known IRE in ferritin mRNA and the 5′-UTR sections of APP RNA is displayed in Figure 4B. These alignments showed that the APP 5′-UTR sequences and the IRE in ferritin-H mRNA shared 70% of their sequences. Several separate transfection experiments were used to evaluate the full functions of this unique iron-sensitive region in the 5′-UTR of APP mRNA [28].

Recently [40], we have predicted the secondary and tertiary structures of the APP IRE RNA, as well as the docking models of the APP IRE RNA complex with IRP1. Prior predictions have been made regarding the ferritin IRE RNA/IRP1 complex [115]. The folded APP RNA secondary structure predictions were based on the 5′-UTRs encoding IRE stem loops. Using thermodynamic studies, the secondary structure of APP IRE is predicted to consist primarily of base-paired stems and hairpin loops [40]. The secondary structure of APP IRE stem loops and the ferritin IRE stem loops are comparable. There are quantitative differences in the interactions of the IRE RNA family with the iron signal and other cellular macromolecules, including the IRP repressor and translation initiation factor activators. The same amount of iron in the same tissue (liver) increased ferritin protein biosynthesis more than mitochondrial aconitase biosynthesis in vivo, which can be explained by the little structural variations in IRE RNAs [116].

In brain tissue, there was more amyloid protein biosynthesis than mitochondrial ferritin and aconitase protein production. A greater proportion of APP mRNA molecules than ferritin mRNA molecules are related to IRP when cellular iron levels are low. When ferritin and mitochondrial aconitase mRNA are compared, there is less APP IRE RNA/protein dissociation [40,41,117], which may be due to differences in the way the various encoded proteins are used metabolically. Because a disproportionately greater number of APP IRE RNA molecules are accessible for initiation factor and ribosome binding than for ferritin and mitochondrial aconitase mRNAs, when IRE RNA/IRP interaction is disturbed by high iron levels, the brain expresses more APP protein. IRP1 binds to the APP IRE stem loop selectively, according to the results of the RNA electrophoretic mobility shift experiment [32]. However, RNA gel shift assays revealed that the mutant APP 5′-UTR cRNA probe is no longer able to bind to IRP [28]. To regulate the iron-dependent translation of APP expression in brain cells, IRP mainly interacts with the APP 5′-UTR. The high binding of an IRP to the APP 5′-UTR suggests that the APP protein is necessary for iron metabolism. Assuming that IRP expression can be functionally connected to APP expression, comparing the relative affinity measurement of IRP as binding partners to the 5′-UTR IRE will provide crucial information for screening new lead compounds that obstruct APP translation.

IRP is the regulatory protein that can identify each configuration of IRE RNA [41,113]. A wide range of RNA–protein complexes are produced by subtle, conserved variations in the IRE structure and sequencing. Each member of the IRE RNA family has a somewhat different RNA–protein complex binding stability. There are physiological consequences to the differences in IRE RNA/IRP stability or instability. APP RNA/IRP binding in a solution has a dissociation constant of 32 nM [40], while ferritin and mitochondrial aconitase IRE RNA/IRP had dissociation values of 14.2 nM and 129 nM under the same conditions [41]. The 30-nucleotide IRE RNA sequences [39,40,41], which are found embedded in hundreds of nucleotide-long mRNAs, were the RNA targets. While some RNA elements, such as the IRE structure found in α-syn mRNA 5′-UTR, were anticipated by computational analysis, it has not been demonstrated that they directly bind to IRPs. Nonetheless, their function in transcriptional regulation has been demonstrated [73].

Using electrophoretic mobility shift experiments, it was demonstrated that certain of these RNA elements, which were identified by immunoprecipitation, have an affinity for binding IRP in vitro [25,32]. Because IRP binding affinities for each unique IRE RNA are quantitatively varied, the fraction of IRE RNA inactivated by IRP binding will differ for each IRE RNA at any given time [118]. Compared to APP IRE RNA, ferritin IRE RNA forms a much more stable complex with IRP. Because of this, ferritin mRNA is more resilient to slight variations in intracellular iron concentration than is the translation of APP, which together form the less stable IRE RNA/IRP complex. The structural distinctions between the APP and ferritin IRE RNAs correlate to functional differences in cell metabolism, since each protein encoded in an IRE RNA requires ferritin for iron concentration activities constantly, but the APP only needs it sometimes.

## 6. The Functional Interaction of Alzheimer’s APP mRNA with IRP1

**Figure 5:**
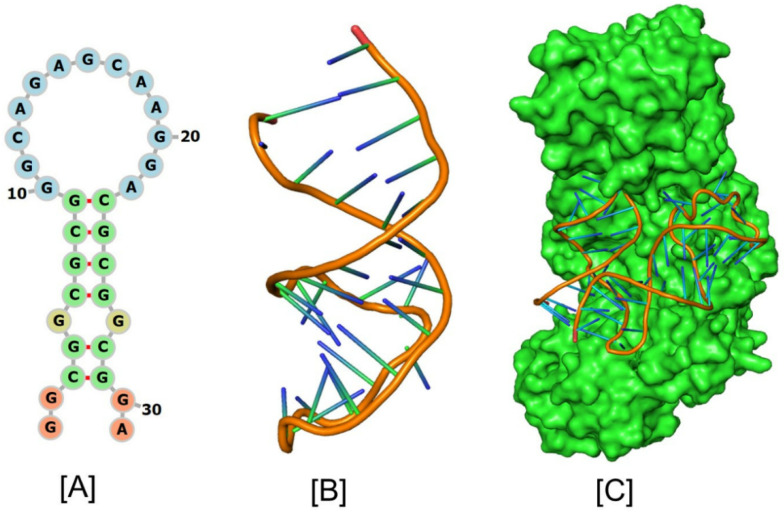
APP iron responsive element RNA structural models. Conserved APP IRE (**A**) secondary and (**B**) tertiary structure. (**C**) APP IRE RNA binding to IRP1. APP IRE RNA: IRP1 complex showing bulge bases and triloop bases flipped out of the helix and making deep contacts in IRP1 protein pockets (modification of figure originally published in ref. [40]).

The IRP (IRP1/IRP2) proteins are recognized by the extremely specialized element called the IRE. That IRP binds strongly to the APP 5′-UTR suggests an integral involvement of APP holoprotein in iron homeostasis. IRPs can control the translational stability or inhibition of mRNAs harboring IREs because of their extremely distinctive recognition pattern for IREs [109,119]. Ferritin translation is induced by the iron-dependent interaction of IRPs with IRE stem loops at the 5′-UTR, which increases the cell’s ability to store iron and shields neurons from oxidative stress [32,120,121]. The synthesis of protein is either boosted or lowered by the interaction of IRE and IRP1, depending on whether the IRE is in the 3′- or 5′-UTR. By regulating the stability of the Tf mRNA through interactions with RNA proteins specific to the 3′-UTR, IRPs regulate the rates at which cells absorb iron [72,122,123]. The C bulge and the AGU apical loop are where the IRP mostly binds to IREs. The structural shape of RNA and its ability to interact with protein molecules define its activity [124]. To identify the nucleotide residues that regulate the IRE RNA conformation and the RNA/protein interaction, the tertiary structure must be predicted. Like ferritin, the APP 5′-UTR mRNA interacts with APP IRE/IRP in response to iron to regulate the expression of APP synthesis [72]. The APP IRE RNA structural model and a typical model of the APP RNA/IRP1 complex are shown in Figure 5A–C [40,117].

Figure 5C shows the precise binding manner of the APP IRE RNA interaction in the IRP1 protein’s binding pocket. The IRE RNA has a bent structure, whereas the IRP1 protein has an L shape. The area enclosed by IRP1 protein domains 1–2 and 4 is where IRE joins the complex.

The APP RNA/IRP1 complex only has two contact sites, which are far apart, to achieve binding selectivity [115,125]. Bonds to the terminal loop and stem disrupting C through two distinct binding sites are the primary way that APP RNA interacts with IRP1 [125]. The complex’s great affinity and selectivity are attributed to the about twenty RNA/protein linkages that are dispersed among the binding sites. A15, G16, and U17 are crucial contacts for identification in the apical loop. Domains 1 and 2 in the globular form blocking the formation of ten connections between amino acids in a pocket created in domain 3 and A15 and G16 in the pseudo-tri-loop at the RNA terminal. In IRP1 domain 4, C8 forms eight bonds with amino acids. The amino acids in IRP1 domain 4 and the stem below C8 are connected in four other ways [115].

Conformational alterations in the RNA and probably the unliganded protein are responsible for the variations in the solution structures of free RNA and protein-bound IRE RNA. A significant portion of the IRE RNA’s surface in the RNA/protein crystal structure is open to interactions with metal ions, other proteins, and RNA [35]. IRP1 most likely uses the same bonding mechanism to bind all naturally existing IRE RNA. Indicating that the APP IRE RNA conformational flexibility may be a contributing factor to the observed affinity differences between these IRE RNA/IRP protein bindings and underscoring the significance of conformational flexibility for this high-affinity binding, the structure of the APP IRE RNA bound to IRP1 deviates from the predominant structure of the RNA in solution. These interactions are necessary for proper translational control of APP mRNA.

Like ferritin RNA binding to IRP1, Alzheimer’s APP IRE RNA binds in the same binding site to IRP1. APP IRE RNA used a collection of the functionally active residues found in the IRP1 binding site to bind IRP1 in a cleft. The APP RNA and IRP1 complex is held together by a multitude of linkages. The region where IRP1’s amino acid residues and RNA terminal make many hydrogen bonds in a pocket created in domain 3 is blocked by domains 1 and 2 in the globular form. During the complex formation process, IRP1’s amino acid residues (Ser127, Arg269, Phe302, Thr438, Asn439, Ser475, Asn535, Arg728, Gly777, Ser778, and Ser780) interact with the APP IRE RNA stem loop by hydrogen bonds [40]. The associated APP RNA is located close to these amino acid residues. The APP RNA/IRP complex is more stable when hydrogen bonds are present. The structural complementarity of APP IRE RNA allowed it to firmly fit into the binding site of the IRP1 protein [40]. The in vivo response to iron levels, where APP, ferritin, mitochondrial aconitase IRE RNAs are present, is where the largest variations in IRP1 binding in solution are observed [40,41,113], and differ by at least an order of magnitude [40,118,126]. Either mobility shift in gel electrophoresis or fluorescence quenching in solution demonstrates that APP, ferritin and mitochondrial aconitase IRE RNA have relative binding affinities with IRP1 that are roughly ten times different. The nanomolar binding affinity [41] for solution fluorescence differs from the picomolar binding affinity [32] resulting from gel shifts. One explanation for this disparity could be because the gels include an adsorptive component.

Ferritin IRE RNA binds IRP1 more firmly than APP and m-aconitase IRE RNA [40,41], suggesting quicker helix bending kinetics during protein binding [39]. Since the RNA contact sites in IRP1 have a conserved structure, changes in the helical structure must be the source of differences in the stability of the RNA/protein complex. When the helices above and below the C bulge rupture or the top stem is lengthened, the binding affinity of IRP1 is changed [73]. Quantitative differences in IRP1 binding affinity and the intensity of the iron response in vivo are linked to natural variations in the helix base pairs of IRE carrying RNAs coding for distinct proteins. The required control of iron homeostasis at the cellular level is provided by the IRE RNA/IRP machinery, which post-transcriptionally modifies target gene expression in accordance with cellular iron levels. As demonstrated for several IRE RNAs in a range of cultured cell types, the iron chelator desferrioxamine (DFO) enhanced IRP1 binding to IRE RNA in cells treated with it [32,127]. Before the current findings, the chemical mechanism by which iron destabilized APP RNA/IRP1 complexes was unclear [40]. Iron selectively weakens the APP RNA/IRP1 binding affinity by four-fold [40], whereas ferritin and mitochondrial aconitase IRE RNA/IRP1 binding decreased by seventeen- and six-fold, respectively, demonstrating the effect of nature’s management of riboregulation among IRE RNAs [41]. The distinct topologies of the RNA/IRP1 sequences determine the extent of iron activity.

**Figure 6:**
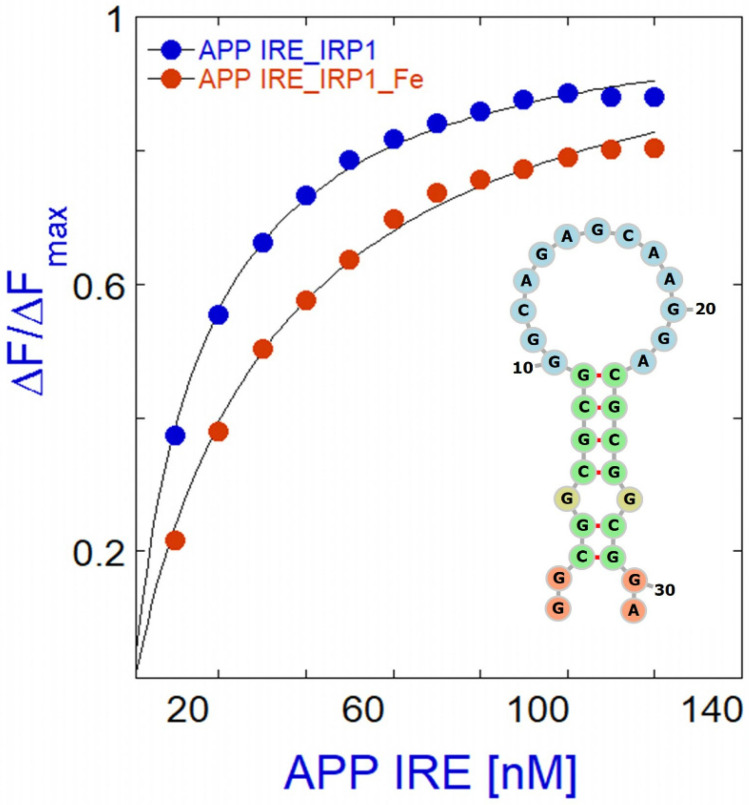
Iron selectively weakens APP IRE RNA/IRP1 interactions. IRP1 binds to the APP IRE RNA with nM affinity in the absence and of iron. Binding curve (protein fluorescence quenching) and conserved IRE secondary structure are prepared from binding data originally published in ref. [40].

Metal ions bind directly to the IRE RNA, according to NMR spectra, ethidium bromide displacement [41,128], and the absence of predicted metal ion binding sites on IRP outside of the [4Fe-4S] cluster insertion site. Additionally, eIF4F binding to ferritin IRE RNA is iron-sensitive [129,130,131]. Iron increased the binding affinity of ferritin IRE RNA with eIF4F [130,131]. Iron lowers APP IRE RNA binding affinity to IRP1 (Figure 6). Therefore, iron causes the binding competition for IRE RNA binding to shift from IRP to eIF4F, even though IRP and eIF4F are in competition with each other for this binding. Ultimately, the complex building process involves several RNA/protein bonds, which increase with the presence of iron for IRE RNA/eIF4F complexes and decrease with the addition of iron for IRE RNA/IRP1 complexes. The repressor protein IRP1 was liberated by iron-induced conformational changes in IRE RNA, and increased binding of the eukaryotic initiation factor eIF4F led to an increase in translation [129,131].

## 7. Translational Control of Alzheimer’s Amyloid Precursor mRNA

**Figure 7:**
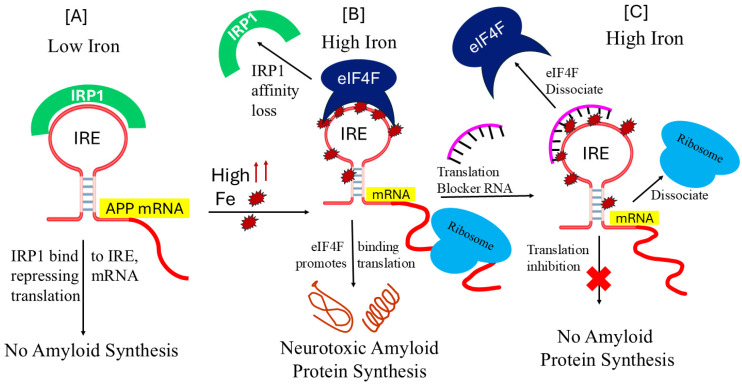
Diagrammatic representation of the translation of the amyloid-β therapeutic target APP IRE RNA. (**A**) IRP can bind to the IRE loop in the 5′-UTR of the APP mRNA and stop amyloid translation when there is a low iron content. (**B**) In addition to allowing eIF4F and ribosome binding to APP mRNA, which overexpresses neurotoxic amyloid precursor protein, high iron levels also dissociate IRP. (**C**) Small-molecule RNA or peptide that binds to APP IRE RNA and inhibits the synthesis of amyloid proteins, hence inhibiting eIF4F and ribosomes.

The 5′-UTR of APP mRNA, like ferritin, controls APP expression with the help of IRP at the level of translation in response to iron, as shown in Figure 7A,B. APP 5′-UTR is unique to the precursor transcript that encodes stable RNA secondary structure, the acute box that regulates 40S ribosome scanning and facilitates the onset of APP synthesis. IRPs regulate the translation of APP and ferritin mRNA as well as the stability of TfR mRNA [132]. IRPs have a potential to dual role as an RNA repressor protein in the absence of iron and promote translation in the presence of iron [30,31]. By controlling iron homeostasis on several levels, these systems optimize iron transport, storage, and regulation at the cellular and systemic levels in vivo. However, neurons are destroyed when there is too much iron in the brain tissues as a result of an imbalance in the iron regulating system [16]. The structure of IRE RNA, which affects the binding of eIF4F and IRP, as well as the IRP protein itself, can be changed by increasing the quantity of iron in cells. IRP1 loses its capacity to bind IRE RNA when it forms a cytosolic aconitase and attaches an iron–sulfur cluster. IRP1 conversion to cytoplasmic aconitase rises and IRP1 availability to bind to IRE RNA falls with increasing iron concentrations and iron–sulfur cluster formation [133]. Consequently, decreasing cellular iron levels alter RNA conformations to decrease IRP binding, increase IRP2 degradation, and decrease RNA/IRP1 binding [134,135]. Iron overload-induced disruption of the IRP/IRE connection can lead to the destabilization of TfR and DMT1 mRNA and the translation of ferritin- and ferroportin-related genes [31,136,137]. Consequently, when iron overload occurs, iron export and storage might rise, while iron absorption is inhibited [121]. Iron homeostasis will be compromised, and AD will develop and worsen because of disruption of the IRP/IRE signaling system.

Protein synthesis begins when eukaryotic initiation factor (eIF4F) attaches itself to the 5′ cap of mRNA. The intricate process of connecting ribosomes necessitates the binding of numerous initiation components and proteins to create an initiation complex that contains ribosomal subunits, initiator tRNA, and mRNA. We showed that APP IRE RNA may bind to eIF4F, which is required for translation initiation, in a preferred and potent manner [132]. Cellular iron levels can be facilitated by eIF4F, since it binds to IRE RNA competitively with IRP, indicating that two proteins occupy the overlapping binding sites [132]. There is still much to learn about the structure of an initiating complex for active protein synthesis. Iron, however, can directly bind to the IRE RNA and change the structure of the mRNA. IRE RNA and eIF4F will interact more readily because of the structural changes in the mRNA caused by iron binding, potentially outcompeting IRE and IRP binding. IRP/IRE signaling pathway disruption will affect iron homeostasis and could contribute to the onset and progression of AD. The process by which cellular iron signals alter IRP affinity for APP IRE RNA was not understood until recently [40]. The physiological iron signal on APP mRNA translation is represented by a model for iron-regulated neurotoxic amyloid protein synthesis (Figure 7B). Iron signals allow ribosome and initiating factor binding and aid in mRNA translation. Conversely, when the IRE is present in the 3′-UTR, it controls target mRNA nuclease binding and mRNA breakdown [30,31]. Rather than controlling nuclease binding, the majority of identified IREs control ribosome binding. IRPs can function as both a translational enhancer and an inhibitor [30,31]. The body struggles to keep each cell’s iron content at the right level.

IRP1 inhibits ribosome binding and the initiation factor eIF4F limits amyloid formation by binding tightly to the IRE RNA control of neurotoxic protein synthesis at low cellular iron levels. Iron can, however, also directly attach to the target mRNA IRE at high cellular iron levels, changing the mRNA conformation. Iron binding causes structural changes in the target mRNA that facilitate interaction between eIF4F, mRNA, and ribosomes and encourage the dissociation of IRP from the mRNA. This complex facilitates the translation of the neurotoxic overproduction of amyloid that leads to the advancement of AD.

## 8. Therapies for Amyloid-β Control That Target APP IRE mRNA

APP mRNA translation is upregulated by iron through 5′-UTR sequences; it is highly feasible that APP 5′-UTR-directed translation blockers provide anti-amyloid efficacy for AD. Through the uniquely folded 5′-UTR of the precursor transcript, APP translation can be selectively targeted by small RNA-based molecules to suppress APP expression [138,139], providing a new target to support current tactics, since the amount of APP translated in response to iron overload in the brain is crucially regulated by the APP 5′-UTR [28,72]. The pathological characteristic of AD is plaque, which is formed when Aβ peptide cleaves from the APP. A method to find and describe inhibitors of this process involves applying knowledge of the translational control circuits through which iron controls translation of the APP and iron storage protein ferritin via interactions with their respective 5′-UTRs, each of which encodes distinct versions of IRE [32]. Consistent with the role of an active IRE in the APP 5′-UTR [32], the iron chelator, desferrioxamine, limited APP expression [140].

Posiphen and phenserine are a new class of small compounds that reduce the development of Aβ in the brain while blocking the APP 5′-UTR IRE RNA-directed translation of APP in astrocytoma and neuroblastoma cell lines and in rats [141]. The small RNA-based molecule strategy depicted in Figure 7C has been demonstrated to function as an inhibitor of APP translation, which may have the ability to prevent amyloidogenic neurodegeneration while enhancing iron balance and counteracting cognitive decline. It has been shown that APP 5′-UTR-directed compounds can be screened for in pharmacological libraries to limit the translation of APP and, eventually, the output of Aβ peptide from neuronal cell culture systems [142]. The APP IRE 5′-UTR folds into a stable RNA secondary structure, and its sequences were discovered to be different from normal eukaryotic mRNAs such as FMR mRNA (fragile X syndrome) similar to ferritin and α-syn 5′-UTR IRE RNA. By inhibiting 40S ribosome translation scanning, the APP IRE RNA raises the baseline translation of reporter mRNA [28] and FMR 5′-UTR sequences are organized and fit the Kozak paradigm. Small molecule-based drugs targeting the APP 5′-UTR IRE-directed inhibitors and high-throughput screening of this RNA target are expected to identify novel therapeutic agents for AD [140]. In neuroblastoma (SY5Y) transfectants, researchers found multiple pharmacological hits that reduced the translation of the hybrid APP RNA 5′-UTR transcript by >95% [142]. Through the 146 nt APP IRE RNA 5′-UTR sequence, several FDA drug classes, including posiphen, phenserine, APP blocker-009, paroxetine and DFO, inhibited the translation of luciferase reporter mRNA [140]. Additionally, without secretase activation, azithromycin inhibited the translation of luciferase reporter mRNA via the APP 5′-UTR sequence [143]. Paroxetine and N-acetyl-cysteine (NAC) were demonstrated to restrict amyloid levels in the TgCRND9 mouse model of AD, establishing proof of concept [144].

Posiphen is a small chemical molecule that blocks the translation of several neurotoxic proteins, such as APP and α-syn, by targeting a conserved regulatory region in the 5′-UTR IREs of their mRNAs [145]. Additionally, it was shown that posiphen and phenserine were genuine APP 5′-UTR mRNA-directed translation inhibitors that enhanced cognition by reducing Aβ formation in vivo. The 5′-UTR of APP mRNA contains uniquely folded IRE sequences that were selectively inhibited by high-throughput screening compounds. This class of drugs is still being explored in clinical trials as an anti-amyloid therapy for AD. Posiphen and phenserine are well-known APP translation inhibitors that show anti-amyloid activity when given long-term in vivo; nevertheless, it is yet unknown how they might affect intracellular iron homeostasis [139,146].

Posiphen showed a comparable IC_50_ for APP 5′-UTR-luciferase expression reduction, at 5 μM, while blocker-9 potency was up to 50 times greater. While posiphen boosted APP 5′-UTR activity by 15%, blocker-9 decreased it by two times at 0.1 μM doses. When compared to posiphen, blocker-9 exhibits anti-amyloid efficacy and is a well-tolerated APP 5′-UTR-directed translation blocker [146,147]. At concentrations as low as 100 nM, blocker JTR-009 successfully reduced the synthesis of APP on SH-SY5Y cells. The APP 5′-UTR-directed blocker-9 only showed cell toxicity at doses greater than 30 μM, although dose-responsive tests verified that the IC_50_ against the APP 5′-UTR was in the 1–100 nM range. The toxicity of blocker-9 is comparable to that of 100 μM posiphen [29]. Blocker-9 was more effective than posiphen at inhibiting APP 5′-UTR-assisted translation. Blocker-9 significantly decreased the formation of toxic Aβ peptide levels [32]. In addition, APP blocker JTR-009 restricted APP and amyloid neuronal levels at picomolar doses [139]. In SH-SY5Y neuroblastoma cells and primary neurons, JTR-009 functioned as an intercalator to stop ribosome attachment to the precursor transcript, which in turn suppressed APP expression without altering iron homeostasis [144]. Therefore, blocker-9 was regarded as an effective anti-amyloid because it was unique to 5′-UTR APP mRNA regions as a translation blocker. Posiphen is a much weaker and less selective blocker than blocker-9 [139].

The potential for chelation therapy to decrease iron-induced ROS and Aβ aggregation makes it a potentially effective treatment approach for AD. DFO reduced the clinical development of AD dementia. The iron chelator DFO dissolved premade β-pleated plaque-like amyloid and stopped the production of β-pleated sheets of Aβ_1-42_. By focusing on the IRE in the APP 5′-UTR mRNA, DFO can also inhibit APP translation and reduce Aβ production through iron chelation [148]. RNA/IRP interactions that translate APP were enhanced by iron chelation with DFO. Furthermore, by targeting SNCA 5′-UTR, paroxetine demonstrated in vivo anti-amyloid efficacy in APP transgenic mice, with prolonged treatment against PD to inhibit synuclein translation of SNCA mRNA [77]. Further studies have demonstrated that the anticholinesterase inhibitor phenserine, an iron chelator, DFO cooperate to decrease translation regulated by APP 5′-UTR IRE [142]. The iron chelator DFO and the anticholinesterase inhibitor phenserine were identified as two “proof of concept” drugs that inhibited APP 5′-UTR directed translation enhancement. Figure 7 illustrates the suggested mechanism of action of the small RNA blocker, which is supported by the discovery that Blocker-9 binds selectively to the APP IRE sequences and not to similar RNA probes encoding the ferritin-H IRE. The models suggest a connection between eIF4F function as an amyloid expressor at elevated iron levels and IRP and eIF4F as an iron-dependent translational regulator. The expression of APP and aggregation of Aβ in brain neurons would therefore be expected to be inhibited by small molecule 5′-UTR mRNA translation blocker of eIF4F and/or ribosome.

## 9. Conclusions and Future Directions

The present set of studies demonstrates the feasibility of downregulating Aβ protein levels by selectively targeting 5′-UTR mRNA transcript with the small RNA molecule. Pharmacological lowering of APP levels is appropriate when associated with anti-amyloid efficacy. The findings’ significance for the etiology and therapeutic approaches of brain iron misregulation in AD has undoubtedly grown in terms of targeting APP mRNA. The present study finds several potential pathophysiological aspects linked to AD, such as metabolic abnormalities of Aβ, hyperphosphorylation of tau protein, iron overload, free radical damage, loss of cholinergic neurons, inflammation, and gene alterations. Reports suggest that there are many routes that contribute to the development of AD, making treatment development difficult. Therefore, drugs that target a single target are unlikely to be very useful in treating AD due to the illness’s complex pathophysiology.

In addition to being the standard therapy for other aging diseases, including heart disease, hypertension and cancer, combination therapies will probably be required to treat AD. It is obvious that new and pertinent targets will also require novel treatments. Because Aβ oligomers cause oxidative stress, synaptic dysfunction, alteration of cell membranes, mitochondrial malfunction, and apoptosis, they are thought to be essential in the pathophysiology of AD. There is still disagreement over the precise mechanism of toxicity caused by iron, fibrillar aggregates, oligomers, or both. Several theories have been put up in recent research, all of which agree that iron-induced Aβ aggregation contributes significantly to AD in one way or another. Thus, a promising strategy is to use small molecule-based inhibitors, such as complement RNA or peptides, to modulate Aβ formation. Although most of the described compounds show lack of selectivity towards Aβ aggregates, cRNA small-molecule modulators are selective, specific, and moderately effective in reducing Aβ aggregation. They are also perfect for modulating Aβ aggregation and blood–brain barrier permeability. Because these modulators may bind to the target selectively and effectively suppress Aβ aggregation, creating a small molecule RNA or peptide-based treatment can be regarded as an effective technique. Through the uniquely folded 5′-UTR of the precursor transcript, selective RNA targeting of APP translation can inhibit APP expression [140], offering a new target to support existing strategies.

The identification and characterization of inhibitors of this process can be accomplished by applying knowledge of the translational control circuits by which iron regulates translation of the APP and ferritin through interactions with their respective 5′-UTR, each of which encodes distinct versions of IRE [32]. Even though 3-D structure is a more popular pharmacological target for proteins, the current generation of RNA therapies, including those based on RNA interference, rely significantly on RNA secondary structure. Because RNA targets are smaller than protein targets, RNA treatments have an advantage over protein-based treatments. Blocker-9, posiphen, phenserine, aducanumab, lecanemab, 10-phenathroline, and yohimbine are examples of small compounds that can attach to particular regions of the IRE mRNA and change the function of mRNA in solution and in cultured human cells to reduce Aβ plaques [95,149,150,151,152]. These findings demonstrated that the small RNA binding molecules’ selectivity for binding to folded target RNA structures in solution was the same as their selectivity for binding to living cells. Finding target areas of the IRE RNA for future drug design studies will be necessary to characterize the iron or cRNA binding site on the IRE. Since they have developed over time, animal IRE RNAs serve as model systems for other 3D mRNAs found in all animals. Though proof of principle data regarding small molecule targeting of mRNA structure have been obtained by IRE RNAs, the possibility of chemically modifying mRNA and protein production in biological systems has not been thoroughly investigated. It will be easier to find new approaches and therapeutic targets for drug discovery and development if the systemic, cellular, and molecular mechanisms of biological aging that preceded and enhance vulnerability to AD are better understood and translated.

## Data Availability

No new data were created or analyzed in this study. Data sharing is not applicable to this article.
